# RNA Network Interactions During Differentiation of Human Trophoblasts

**DOI:** 10.3389/fcell.2021.677981

**Published:** 2021-06-03

**Authors:** Tianjiao Chu, Jean-Francois Mouillet, Zhishen Cao, Oren Barak, Yingshi Ouyang, Yoel Sadovsky

**Affiliations:** ^1^Department of Obstetrics, Gynecology and Reproductive Sciences, Magee-Womens Research Institute, University of Pittsburgh School of Medicine, Pittsburgh, PA, United States; ^2^Department of Microbiology and Molecular Genetics, University of Pittsburgh School of Medicine, Pittsburgh, PA, United States

**Keywords:** trophoblast, differentiation, hypoxia, lncRNA, miRNA, gene ontology, RNA network

## Abstract

In the human placenta, two trophoblast cell layers separate the maternal blood from the villous basement membrane and fetal capillary endothelial cells. The inner layer, which is complete early in pregnancy and later becomes discontinuous, comprises the proliferative mononuclear cytotrophoblasts, which fuse together and differentiate to form the outer layer of multinucleated syncytiotrophoblasts. Because the syncytiotrophoblasts are responsible for key maternal-fetal exchange functions, tight regulation of this differentiation process is critical for the proper development and the functional role of the placenta. The molecular mechanisms regulating the fusion and differentiation of trophoblasts during human pregnancy remain poorly understood. To decipher the interactions of non-coding RNAs (ncRNAs) in this process, we exposed cultured primary human trophoblasts to standard *in vitro* differentiation conditions or to conditions known to hinder this differentiation process, namely exposure to hypoxia (O_2_ < 1%) or to the addition of dimethyl sulfoxide (DMSO, 1.5%) to the culture medium. Using next generation sequencing technology, we analyzed the differential expression of trophoblastic lncRNAs, miRNAs, and mRNAs that are concordantly modulated by both hypoxia and DMSO. Additionally, we developed a model to construct a lncRNA-miRNA-mRNA co-expression network and inferred the functions of lncRNAs and miRNAs via indirect gene ontology analysis. This study improves our knowledge of the interactions between ncRNAs and mRNAs during trophoblast differentiation and identifies key biological processes that may be impaired in common gestational diseases, such as fetal growth restriction or preeclampsia.

## Introduction

The fusion of mononucleated cytotrophoblasts into multinucleated syncytiotrophoblasts is a central process in human trophoblast differentiation. Early in pregnancy, this fusion process is a part of the pre-lacunar and lacunar stages of implantation on days 6–12 after fertilization in human pregnancy (Boyd and Hamilton, 1970). Once villi are formed, the fusion of mononucleated cytotrophoblasts into overlying multinucleated syncytiotrophoblasts at the villous surface is accompanied by a dramatic change in cell morphology, transcriptional output, and the production of growth factors and endocrine signals (Sadovsky and Jansson, 2015). Located at the surface of human placental villi, the syncytiotrophoblasts are uniquely positioned to regulate key functions of the placenta in terms of maternal-fetal gas exchange, the uptake of nutrients into the feto-placental compartment, the release of waste to the maternal blood, the production of hormones and the immune and mechanical protection of the developing fetus (Sadovsky and Jansson, 2015; Burton et al., 2016). The subjacent mononucleated cytotrophoblasts, which form a continuous layer early in pregnancy, later become a discontinuous layer of interspersed cytotrophoblasts that function as progenitors for replenishment of damaged or dead syncytium and homeostatic preservation of this critical layer (Jones and Fox, 1991). The syncytiotrophoblast exhibits polarity, with a microvillous plasma membrane facing the maternal blood on the apical side and a basal plasma membrane located adjacent to the cytotrophoblasts and the basement membrane. Considering its functions, it is not surprising that the syncytiotrophoblast microvillous membrane, which interfaces directly with the maternal blood, harbors receptors for diverse plasma proteins, growth factors, immunoglobulins, and other soluble ligands, all linked to intracellular trophoblast signaling cascades (Dearden and Ockleford, 1983; Sadovsky and Jansson, 2015).

Syncytium formation prior to 10–13 weeks of human pregnancy takes place in a hypoxic environment (Burton et al., 2002; Jauniaux et al., 2006; Burton and Jauniaux, 2018). Beyond that point, and once the maternal blood begins to perfuse the intervillous space, hypoxia may be harmful for proper placental function, leading to cytotrophoblast proliferation, attenuated fusion of cytotrophoblasts into syncytiotrophoblasts, reduced hormone and other biosynthetic functions, and overall syncytial damage and trophoblast death, leading to placental injury and diseases such as fetal growth restriction (Fox, 1970; Arnholdt et al., 1991; Pardi et al., 1993; Alsat et al., 1996; Levy et al., 2000; Pardi et al., 2002; Cartwright et al., 2007; McCarthy et al., 2007; Simon and Keith, 2008; Schoots et al., 2018). Seeking to characterize gene expression changes that define trophoblast differentiation, researchers have focused on the effect of hypoxia on the expression of protein-coding genes in term trophoblasts (Roh et al., 2005; Oh et al., 2011; Wakeland et al., 2017; Kwak et al., 2019).

Recent progress in untangling the complexity of the RNA world has shed light on diverse non-coding RNAs (ncRNAs) that play an essential role in shaping cellular differentiated functions. Among these RNAs, microRNAs (miRNAs) and long non-coding RNAs (lncRNAs) represent the two best characterized classes and perhaps those with the most important regulatory potential. Over 2000 miRNAs are encoded in the human genome. Most of these miRNAs act in the cytosol, where they target mRNAs though imperfect base-pairing to block their translation and accelerate their decay (Bartel, 2009). However, despite their relative simplicity, the full impact of miRNAs on gene expression remains incompletely understood. lncRNAs are more diverse, with an estimated 30,000–100,000 nuclear and cytoplasmic species expressed from the human genome (Iyer et al., 2015; Hon et al., 2017; Uszczynska-Ratajczak et al., 2018; Carlevaro-Fita and Johnson, 2019). Further, the action of lncRNAs is complex, spanning interactions with DNA, RNA, and proteins and involving 3D structural flexibility that enables protein scaffolding and the assembly of multi-subunit complexes and nuclear condensates that shape transcriptional and posttranscriptional functions (Carlevaro-Fita and Johnson, 2019; Statello et al., 2021).

Recent discoveries within the field of placental biology highlighted the putative role of lncRNAs and miRNAs in trophoblastic gene regulatory networks, their role in trophoblast differentiation and in response to hypoxic injury, and the impact of these processes on clinically relevant placental diseases (Canfield et al., 2019; Sheng et al., 2019; Saha and Ain, 2020). A systematic inquiry into network interactions of lncRNAs, miRNAs, and mRNAs in differentiating primary human trophoblasts (PHT cells) is lacking. Here, we used an *in vitro* model of cultured PHT cells to investigate harmonized changes of lncRNAs, miRNAs, and mRNAs during PHT cell differentiation. In addition to exposure of PHT cells to hypoxia (Nelson et al., 1999), diverse chemicals and culture conditions have been employed *in vitro* to modulate the differentiation of PHT cells. *Douglas et al.* found that cytotrophoblasts exposed to 1.5% DMSO retain their mononuclear morphology, culminating in drastic inhibition of hCG production (Thirkill and Douglas, 1997). Other approaches to limit trophoblast differentiation include the use of colchicine, an inhibitor of microtubule polymerization (Douglas and King, 1993), cobalt chloride, a hypoxia-mimicking agent (Daoud et al., 2005; Rimon et al., 2008), and the use of Ham’s/Waymouth medium (Douglas and King, 1990; Chen et al., 2004; Bildirici et al., 2018). The deployment of these culture conditions led to the discovery of a repertoire of genes that have been implicated in trophoblast differentiation and to which hypoxia-induced placental injury has been attributed (Jiang and Mendelson, 2005; Chen et al., 2006; Soares et al., 2017).

We sought to hinder trophoblast differentiation using hypoxia or the addition of DMSO to the culture medium, two approaches that have led to reproducible results in our laboratory (Nelson et al., 1999; Yusuf et al., 2002; Roh et al., 2005; Oh et al., 2011; Mouillet et al., 2013; Bildirici et al., 2018; Beharier et al., 2020). We used next generation sequencing technology to identify differentially expressed lncRNAs, miRNAs, and mRNAs during these processes. Importantly, we interrogated the interactions among these RNAs and inferred the main biological processes represented by co-expression patterns.

## Materials and Methods

### Placentas and Dispersed Primary Human Trophoblasts

All placentas used in our studies were obtained from uncomplicated pregnancies and term deliveries at Magee-Womens Hospital in Pittsburgh, under a protocol that was approved by the Institutional Review Board at the University of Pittsburgh. PHT cells were isolated using the trypsin-DNase-dispase/Percollx method as described by Kliman et al. (1986), with modifications as we previously published (Nelson et al., 1999; Mouillet et al., 2010). PHT cells were cultured in DMEM (Sigma-Aldrich, St. Louis, MO) containing 10% bovine growth serum (HyClone, Logan, UT) and 1% P/S antibiotics (Sigma-Aldrich) at 37°C in a 5% CO_2_-air atmosphere, until culture conditions were modified as below.

**FIGURE 1:**
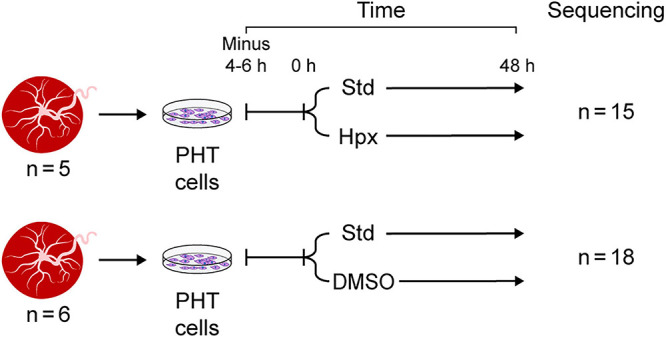
Experimental design. Note that PHT cells were sequenced at 0 h (4–6 h after plating, serving as control) and at 48 h in standard culture conditions or in the two experimental exposure sets: hypoxia (Hpx) or DMSO added to the culture medium.

The data used for this study were derived from two independent sets of experiments, each including several paradigms (Figure 1). In the first experimental set, PHT cells from five independently collected placentas were first cultured for 4–6 h in standard conditions (20% O_2_) to allow adhesion, using protocols established in our lab. Some of the cells were harvested at the end of the initial incubation period (time 0 in Figure 1) and were used as control. The remaining plates from the same culture were maintained for an additional 48 h in either standard culture conditions or in hypoxia (O_2_ < 1%), using a dedicated hypoxia chamber, as we previously described (Mouillet et al., 2010). In the second experimental set, PHT cells from six independently collected placentas were first cultured for 4–6 h in standard conditions as above. The culture continued for an additional 48 h, with some of the cells exposed to DMSO 1.5% (Sigma-Aldrich), designed to mitigate cell differentiation as previously shown (Douglas and King, 1990) and reproducibly validated by us (Schaiff et al., 2000; Yusuf et al., 2001).

### RNA Extraction, Library Preparation, and Sequencing

Total RNA was isolated from from placental specimens by using TRI Reagent (Sigma) according to the manufacturer’s instructions and purified using EconoSpin spin columns (Epoch Life Science, Missouri City, TX). The quantity and quality of total RNA was determined with a NanoDrop 1000 spectrometer (Thermo Fisher, Waltham, MA) and an Agilent bioanalyzer (Agilent Technologies, Santa Clara, CA). From each extracted RNA sample, we used 10 μg of total RNA to generate two types of libraries—one for long RNAs (≥200 nt), including mRNAs and lncRNAs, and one for small RNAs. The libraries were prepared and sequenced by Ocean Ridge Biosciences (Palm Beach Gardens, FL) and by McGill University’s Génome Québec Innovation Centre (Montréal, Canada). For the second set of experiments, the libraries were prepared and sequenced at the Health Sciences Sequencing Core at Children’s Hospital of Pittsburgh. The miRNA samples were sequenced using the QIAseq miRNA sequencing protocol, which links a unique molecular identifier (UMI) to each miRNA to reduce sequencing bias. Data from all experiments were deposited to the Sequence Read Archive (SRA) at the National Center for Biotechnology Information with BioProject IDs: PRJNA674312, PRJNA674329, PRJNA674366, PRJNA704383, PRJNA704399, PRJNA704393.

### Reverse Transcriptase and Quantitative PCR (RT-qPCR)

RNA was extracted from cells with TRI Reagent (Sigma). cDNA was synthesized from 1 μg of total RNA by using the High-Capacity cDNA Reverse Transcription kit (Applied Biosystems, Foster City, CA) according to the manufacturer’s protocol. Template cDNA was PCR-amplified with the gene-specific primer sets (Supplementary Figure 1B). RT-qPCR was performed using SYBR Select (Applied Biosystems) in a ViiA 7 system (Applied Biosystems). Analysis of qPCR data was performed using the delta-delta Ct method (Livak and Schmittgen, 2001), normalized to glyceraldehyde-3-phosphate dehydrogenase expression.

### RNAseq Data Processing

The long RNA libraries were aligned to human reference genome GRCh38 using STAR (2.5.2b), an RNAseq alignment tool (Dobin et al., 2013), and annotated with GENCODE v25 (Frankish et al., 2019). The number of reads per gene was calculated for each RNAseq library, using STAR. We used the definition of lncRNA, which includes antisense RNA, sense intronic RNA, processed transcripts, and sense-overlapping RNAs (Derrien et al., 2012). The 15 small RNA libraries from the first set of experiments were analyzed using our validated miRNA sequencing data analysis pipeline (Chu et al., 2015). Briefly, after pre-processing the library reads, including the removal of adaptor sequences and dimerized primer sequences, we used Bowtie to align all remaining reads with of least 15 nucleotides to the human reference genome (GRCh38) (Langmead et al., 2009). The BEDTools program was used to intersect the alignments to a mature miRNA database maintained by miRBase (v21). The intersected alignments were summarized to obtain counts for all miRNAs. The 18 small RNA libraries from the second set of experiments were processed using the online Qiagen Primary QIAseq miRNA quantification tool^1^ to handle the reads with UMIs. We found an extremely high correlation between miRNA counts with distinct UMIs and total miRNA reads. To ensure consistency across the two experimental sets, the total miRNA reads were used as the miRNA expression data.

The counts of long and short RNAs in the sequencing libraries were assumed to follow negative binomial distributions. The negative binomial test, implemented in the Bioconductor R package DESeq2 (Love et al., 2014), was used to identify differentially expressed lncRNAs, miRNAs, and mRNAs. Fisher exact test was used to perform gene ontology analysis and identify the biological processes that were over-represented in selected mRNAs. All *p*-values from multiple simultaneous multiples tests were adjusted using Benjamin and Hochberg’s method to control the false discovery rate (Benjamini and Hochberg, 1995).

### Model-Based Co-expression Analysis

We proposed a model-based method to identify mRNAs that were co-expressed with lncRNAs and/or miRNAs. Specifically, we assume that the two genes are co-expressed if their expressions Y and X are related through a (generalized) linear model:

g(E[Y|X,Z])=aX+BZ,

where *g* is the link function, Z is the vector of confounding variables, B is a row vector representing the coefficients of Z, and *a* is a non-zero coefficient representing the co-expression relation between X and Y. We then examined whether X and Y were co-expressed by testing if *a* = 0. Note that when X, Y, and Z have a joint multivariate normal distribution, this is equivalent to testing whether X and Y have a zero partial correlation, given Z. As we used a negative binomial model to analyze the gene expression, Y is the count for one gene and X the log-transformed and normalized expression of the other gene. The vector B accounts for the experimental exposure and for placentas used for each batch of PHT cells.

### Analysis of Gene Ontology

Because the functions of lncRNAs and miRNAs are not available from the gene ontology database, we proposed an indirect approach through the gene co-expression analysis described above. Specifically, given a set of lncRNAs or miRNAs and using the model-based co-expression analysis, we identified all mRNAs that are co-expressed with each of the lncRNAs or miRNAs. The top-ranked mRNAs were then used for analysis of gene ontology to identify the enriched biological processes, attributed to altered expression of the set of lncRNAs or miRNAs.

The statistical analyses, detailed above, were performed using R (R Development Core Team, 2012) and Bioconductor (Gentleman et al., 2004). For analysis of lncRNA by RT-qPCR, the fold-change data were analyzed using Kruskal Wallis non-parametric test, with *post hoc* Tukey test for all pairwise comparisons. RT-qPCR data analysis was performed using Prism software (GraphPad Software, San Diego, CA).

## Results

**FIGURE 2:**
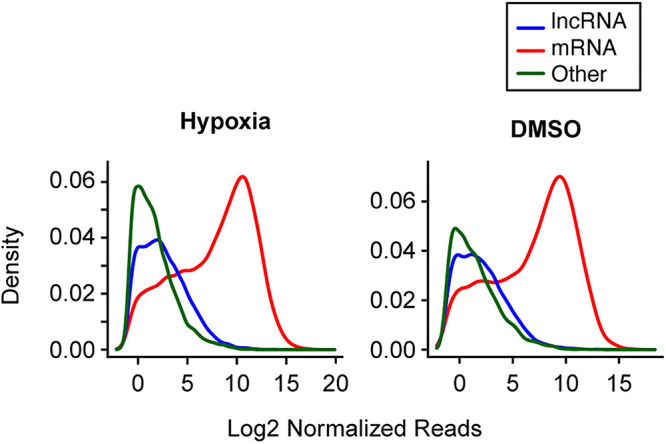
The distribution of selected long RNA reads in PHT cells in the two experimental sets. The *X*-axis represents the average log2 normalized reads per library, and the *Y*-axis represents the estimated probability density. Note that analysis of multiple small RNA species was not performed, as the sequencing procedure was designed to enrich for miRNA (which represented 72.3% of small RNAs in hypoxia and 68.8% of RNAs in DMSO).

Using an average read of 0.5 per library as a cutoff in the first set of experiments, we identified 33,719 long RNAs, including 8,035 lncRNA and 17,298 mRNA species, from the 15 long RNA libraries and 923 miRNAs from the 15 small RNA libraries. In the second set of experiments, we identified 30,421 long RNAs from the 15 long RNA libraries, including 6,988 lncRNA and 16,723 mRNA species, and 2,416 miRNAs from the 15 small RNA libraries. Overall, mRNAs had a higher read count per library than lncRNAs (Figure 2). For example, in the first experiment the median of average lncRNA reads per library was 6.2, compared to 457.5 reads for the mRNAs. In the second experiment, the corresponding values were 4.2 and 267.7, respectively. We also used RT-qPCR to validate the expression changes of 10 lncRNAs (Supplementary Figure 1), as we previously did for mRNA and miRNA transcript data (Mouillet et al., 2010; Xie et al., 2014).

**FIGURE 3:**
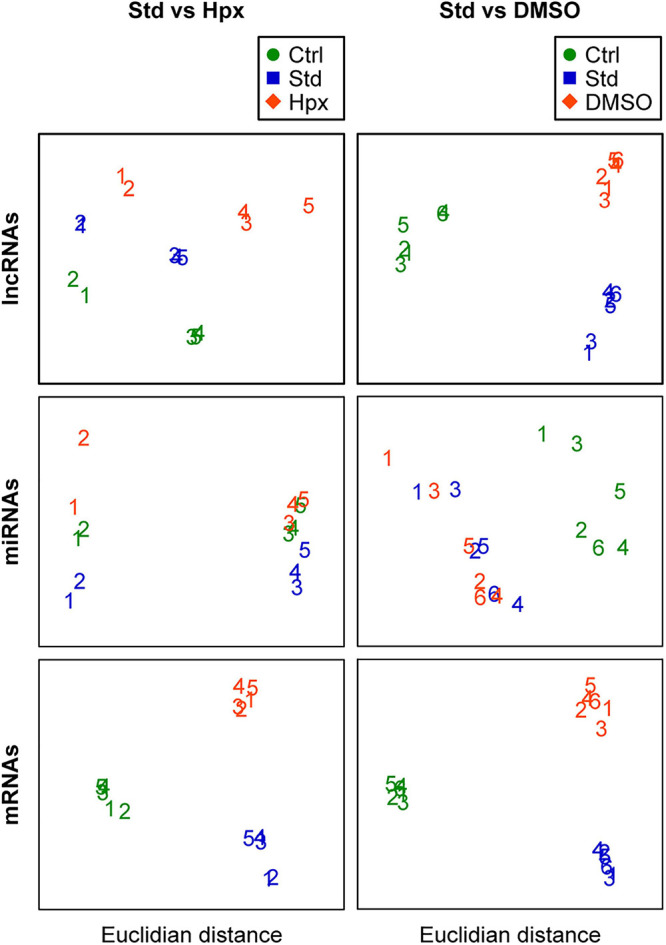
Multidimensional scaling plots of RNAs in the two experimental sets. Classic multidimensional scaling plots of lncRNAs, miRNAs, and mRNAs were created using Euclidean distance as dissimilarity matrix across the samples. The cells were sequenced after plating (Ctrl), 48 h after culture in standard condition (Std) or after culture in hypoxia (Hpx) or in medium with added DMSO. The numbers in the plots represent each placental ID in the two exposure sets.

To visualize the effect of exposure and potential confounding factors on the PHT transcriptome, we normalized and log transformed the long RNA and miRNA library data, using the regularized and variance-stabilizing transformation method in the Bioconductor package DESeq2. We then performed classical multidimensional scaling, respectively, for lncRNAs, miRNA, and mRNAs (Figure 3). These plots showed that the experimental conditions markedly influenced the PHT transcriptome. The exposure effect dominated for mRNAs in the two experimental sets and for lncRNAs in the second set and was clearly visible for lncRNAs in the first set and for miRNAs in both experimental sets. The plots also showed a batch effect on the expression of lncRNAs and miRNAs in the first experimental set (hypoxia), as samples 1 and 2 were processed by a different lab than samples 3–5. Our data also suggest an effect of the placenta on miRNAs in both experimental sets.

**FIGURE 4:**
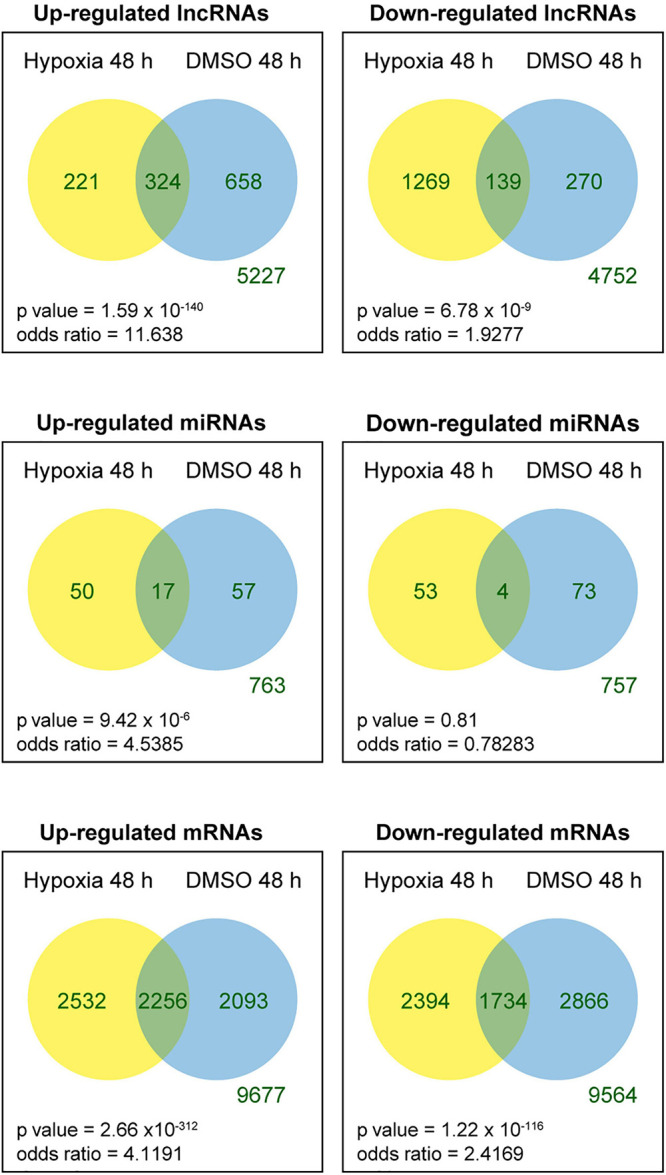
Venn diagram of differentially expressed RNAs across the two experimental sets. Fisher exact tests were used to determine whether the genes that were up- or downregulated in one experimental set were more likely to be regulated in the same direction in the other experimental set.

**FIGURE 5:**
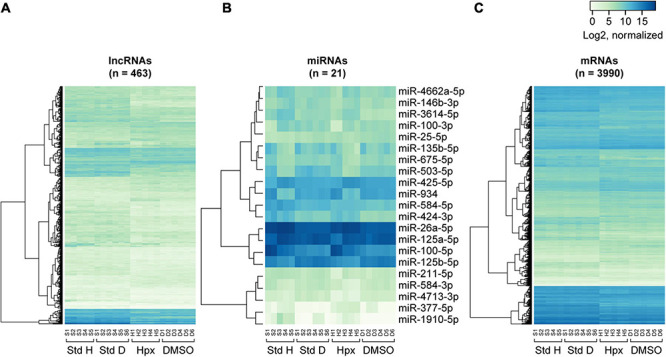
Heatmaps of RNA expression clustering across the experimental paradigms. The heatmaps were constructed on the basis of normalized log2 gene expression for concordantly differentially expressed lncRNAs **(A)**, miRNAs **(B)**, and mRNAs **(C)** in the two exposure sets. Note that the first letter of the sample labels at the bottom of each plot represent experimental condition: “S” for 48 h in standard conditions, “H” for 48 h in hypoxic conditions, and “D” for 48 h in DMSO-containing medium. The number represents placental ID in each experiment. “Std H” and “Std D” refer to 48 h Std conditions in the hypoxia or DMSO experiments, respectively.

We tested the lncRNAs, miRNAs, and mRNAs that were differentially expressed between the conditions in the two experimental sets. For this, we used negative binomial regression models with two factors: the exposure factor (hypoxia, DMSO) and the placenta factor, which refers to inter-individual variation in placental transcriptome and possible differences in processes related to placental collection and cell preparation. After controlling the false discovery rate at 0.05, the numbers of differentially expressed lncRNAs, miRNAs, and mRNAs are shown in Table 1. For convenience, herein genes differentially expressed under the hypoxic as opposed to the standard condition are termed “hypoxia-DE” genes, and genes differentially expressed with DMSO as distinguished from the standard condition are termed as “DMSO-DE” genes. We next examined the number of lncRNAs, miRNAs, and mRNA that exhibited concordant (Figure 4) or discordant (Supplementary Figure 2) expression change across the two experimental sets. We noticed a significant concordance (up or down) in RNA expression change between the two sets of experimental exposures, namely “hypoxia-DE” and “DMSO-DE” genes (Figure 5). This concordance was noted primarily for lncRNA and mRNA species (Figures 5A,C), with less concordance for up- or downregulated miRNAs (Figure 5B).

**TABLE 1:** The numbers of differentially expressed RNA transcripts in the two experimental sets.

	**Std vs. Hypoxia**		**Std vs. DMSO**	
	**Up**	**Down**	**Up**	**Down**
mRNA	4,803	4,236	4,354	4,607
lncRNA	571	1,658	1,012	422
Other RNA	311	1,234	520	264
miRNA	69	58	94	156

We also assessed the similarity of the two experimental sets by examining expression differences in mRNAs, lncRNAs, and miRNAs, between the 0 and 48 h time points, under standard conditions. We found an extremely high concordance (up or down) in the expression change of all three types of RNAs between the two experimental sets (Supplementary Figure 3). This confirms that the two experimental sets are comparable for similar conditions, thus supporting our experimental approach.

We provided additional support to our findings by comparing our gene expression changes with published human placental single cell RNAseq (scRNAseq) data, predicting that our data would be similar to gene expression changes between syncytiotrophoblasts and cytotrophoblasts. We performed this analysis using the PlacentaCellEnrich tool (Jain and Tuteja, 2021), available for protein coding genes, and examined changes among the 19 mRNAs with the highest concordant log2-fold increased expression and the 6 mRNAs with the highest concordant log2-fold decreased expression between standard conditions and hypoxia/DMSO. Among the selected 25 mRNAs (Supplementary Table 1) the vast majority of our results were consistent with the scRNAseq data, as predicted, with expression changes between standard conditions vs. hypoxia/DMSO that are similar to changes between syncytiotrophoblasts and cytotrophoblasts. Notably, discrepancies were more common for one of the databases, likely reflecting much lower mRNA expression levels and a lower magnitude of the log2-fold change for mRNAs that are downregulated during cell differentiation.

To define pathways that might be implicated in reduced trophoblast differentiation, we performed direct and indirect gene ontology analyses. We focused on genes with at least a moderate expression, defined as having a minimum of 25 reads per library. For mRNA, we focused on 3,338 hypoxia-DE genes, 2,232 DMSO-DE genes, and 988 DE genes that were concordantly altered in the two sets of experiments. Table 2 shows the shared biological processes that were significantly enriched based on mRNA changes in “hypoxia-DE” and in “DMSO-DE” transcripts, irrespective of lncRNA or miRNA co-expression. Supplementary Table 2 shows differentially expressed mRNAs that were concordantly expressed in the two exposure sets and that are co-expressed with at least one differentially expressed lncRNA and miRNA, where both lncRNA and miRNA exhibit concordant expression change (up- or downregulation) between the two exposure sets.

**TABLE 2:** Biological processes enriched in mRNAs that exhibited a concordant expression change during trophoblast differentiation^*a*^ and ranked by adjusted *p*-value.

**Process**
Immune response
Female pregnancy
Signal transduction
Inflammatory response
Cell surface receptor signaling pathway
Immune system process
Chemokine-mediated signaling pathway
Cell-cell signaling
Response to drug
Retinoid metabolic process
Cytokine-mediated signaling pathway
Positive regulation of cytosolic calcium ion concentration
Positive regulation of T cell proliferation
Defense response
Regulation of calcium ion-dependent exocytosis
Calcium ion-regulated exocytosis of neurotransmitter
Multicellular organism development
Cell adhesion
Positive regulation of phagocytosis, engulfment
Chemotaxis
Single organismal cell-cell adhesion
Innate immune response
Keratinization
Cellular response to lipopolysaccharide
G-protein coupled receptor signaling pathway
Positive regulation of ERK1 and ERK2 cascade
Positive regulation of cell proliferation

*^a^Trophoblast differentiation was defined as expression change in control vs. hypoxia and in control vs. DMSO exposure.*

**FIGURE 6:**
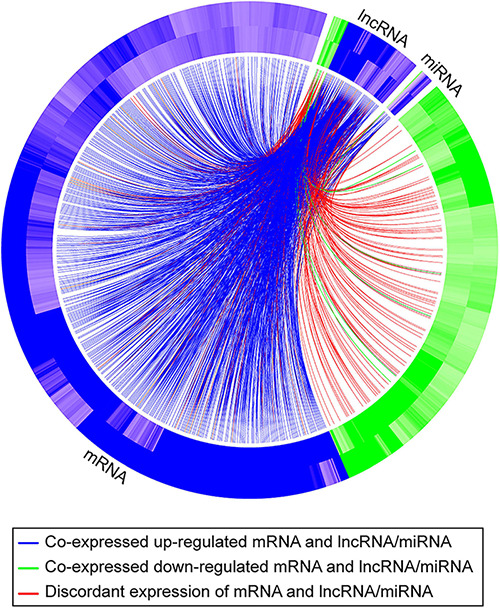
A Circos plot depicting the association of mRNAs, lncRNAs, and miRNAs that exhibit a concordant expression change in the two exposures (hypoxia and DMSO). The outer band of the ring represents log2 fold changes in standard vs. hypoxia exposure, and the inner band of represents log2 fold changes in standard vs. DMSO exposure. Blue denotes that the RNA is upregulated; green denotes that the RNA is downregulated. The curved lines connect each lncRNA/miRNA to the co-expressed mRNAs, with blue (upregulation) and green (downregulation) lines representing positive correlations and red lines representing negative correlations.

To further pursue the biological pathways reflecting mRNA expression changes that were associated with differentially expressed lncRNAs and/or differentially expressed miRNAs, we performed indirect gene ontology analysis as described in section “Materials and Methods.” We first selected the 438 hypoxia-DE lncRNAs and 85 hypoxia-DE miRNAs, 167 DMSO-DE lncRNAs and 115 DMSO-DE miRNAs, as well as 66 concordant DE lncRNAs and 9 concordant DE miRNAs. We then performed model-based co-expression analysis for these associations on the basis of our negative binomial regression model. Table 3 shows biological processes attributed to mRNAs that were expressed in a concordant manner in the two exposures, in association with changes with either lncRNA *or* with miRNA in the same conditions. Lastly, we identified the biological processes attributed to mRNAs that were differentially expressed in a concordant manner between the two exposures in association with changes with both lncRNA *and* with miRNA in the same conditions (Table 4). These associations are also shown in the Circos plot (Figure 6).

**TABLE 3:** Biological processes enriched in mRNAs that were associated with lncRNA *or* miRNA during trophoblast differentiation, and ranked by adjusted *p*-value.

**Association**	**Biological processes**
mRNA associated with lncRNA^*a*^	Positive regulation of transcription from RNA pol II promoter
	Response to drug
	Inflammatory response
	Response to mechanical stimulus
	Positive regulation of gene expression
	Cellular oxidant detoxification
mRNA associated with miRNA^a^	Female pregnancy
	Cell adhesion
	Inflammatory response
	Extracellular matrix disassembly
	Cell-cell signaling
	Positive regulation of angiogenesis
	Response to drug

*^a^At least one DE lncRNA or one DE miRNA, respectively, were associated with mRNA in the two exposures.*

**TABLE 4:** Biological processes enriched in mRNAs that were associated with lncRNA *and* miRNA during trophoblast differentiation^a^, and ranked by adjusted *p*-value.

**Process**
Female pregnancy
Cholesterol metabolic process
Lipoprotein metabolic process
Positive regulation of cytosolic calcium ion concentration
Placenta development
Cell adhesion
Inflammatory response
Positive regulation of phagocytosis, engulfment
Retinoid metabolic process
Cytokine-mediated signaling pathway
Cellular response to hormone stimulus
Signal transduction
Immune response
Chemotaxis
Response to drug

*^a^At least one DE lncRNA and one DE miRNA were associated with altered mRNA expression in the two experimental exposures.*

## Discussion

To decipher RNA interactions during differentiation of primary term human trophoblasts, we interrogated two sets of experiments, where the normal differentiation process was hindered either by the physiologically relevant exposure to hypoxia or by using the chemical DMSO (Thirkill and Douglas, 1997; Nelson et al., 1999; Yusuf et al., 2002; Roh et al., 2005; Oh et al., 2011). Because each of these exposures may influence the PHT transcriptome in a manner that is independent of the effect on differentiation, we focused on RNA interactions that are shared by both exposures. We surmised that identification of such interactions may suggest RNA regulatory pathways that govern trophoblast differentiation.

Our informatics-based data offered general insight into changes in the trophoblast transcriptome during differentiation. We noticed that the average number of reads for each lncRNA is about 1.3∼1.5% of the average mRNA read number. Based on Ensembl Gene 87 (Aken et al., 2017), the average length of a lncRNA molecule is 1 kb, and 1.7 kb for mRNA. Therefore, the average expression of lncRNA in PHT cells is between 2.2 and 2.5% of mRNA. This lower expression of lncRNA relative to mRNA was shown in other cell systems (Palazzo and Lee, 2015). Note that the reads per library for lncRNAs and mRNAs in the first experimental set are higher because of the larger average library size in that set. Further, our data show that the distribution of the reads per library of “other RNAs” is somewhat different between the two experiments (Figure 2). These two factors likely reflect differences in library size, preparation, and sequencing technology between the two experimental sets. Indeed, even among the “other RNAs,” these factors tend to have a greater effect on smaller RNAs (Supplementary Figure 4). Importantly, these technical factors would not affect our analysis, because we tested the differential expression in the two experimental sets separately, and took library size into account in our statistical tests.

Importantly, we found that lncRNAs, miRNAs, and mRNAs respond to hypoxia and DMSO in different ways. Notably, mRNAs and lncRNAs exhibited a similar change in expression patterns despite of their vastly different expression levels: both RNA types clustered by the experimental conditions (Figure 3), both differentially expressed mRNAs and lncRNAs tended to have larger log2 fold expression change (Figure 4), and both exhibited significant concordance in DE transcripts between the two experimental sets (Figure 5). In contrast, DE miRNA clustering reflected not only the experimental exposure but also the placenta used for PHT cell dispersal and, in the case of hypoxia, even the laboratory performing the sequencing Figure 3). This observation could be explained by the smaller log2 fold expression change in differentially expressed miRNAs (Figure 4), a smaller number of miRNA with a concordant expression change between the two experimental sets (Figure 5), or differences in sequencing technology among the laboratories.

We identified mRNAs that were concordantly differentially expressed across both experimental sets and correlated with altered expression of ≥ 1 lncRNA *and* miRNA (Supplementary Table 1). Some of the identified transcripts were previously shown to be related to germane trophoblast processes and to complications of pregnancy. SDC1 (Syndecan1) was shown to have a positive correlation with trophoblast differentiation and exhibited lower expression in hypoxia/DMSO exposure compared to standard conditions (Prakash et al., 2011). Several identified differentially expressed mRNAs are known to regulate the invasion or adhesion of trophoblasts and other types of cells. KISS1 has a role in early placentation and implantation, ADAM12 (ADAM metallopeptidase domain 12) was shown to control trophoblast fusion through E-cadherin, and NECTIN3 (Nectin Cell Adhesion Molecule 3) is a member of a cell adhesion family of proteins (Reymond et al., 2000; Aghababaei et al., 2015; Hu et al., 2019). Some of the genes with the most pronounced expression changes are related to preeclampsia, where hypoxia is commonly implicated in disease pathogenesis (Tal, 2012). Indeed, reduced placental expression of LGALS13 (Galectin 13), a member of the glycan-binding proteins that regulate innate and adaptive immune responses, was found in women with preeclampsia (Than et al., 2014). Similarly, the expression of SDC1, NPPB (natriuretic peptide B), GDF15 (growth differentiation factor 15), and ADAMTS6 (ADAM metallopeptidase with thrombospondin type 1 motif 6) all had a lower expression in our experimental exposures, and all are lower in preeclampsia (Junus et al., 2014; Chen et al., 2016; Gandley et al., 2016; Jiang et al., 2020). In contrast, our data are inconsistent with respect to the expression of PAPPA2, a regulator of trophoblast invasion and migration, or MNDA (myeloid cell nuclear differentiation antigen), which exhibited a lower expression level in our paradigms but is elevated in placentas from women with preeclampsia (Wagner et al., 2011; Kolialexi et al., 2017; Neuman et al., 2020).

A major innovative aspect of our work is the use of the model-based co-expression analysis. The Pearson correlation coefficient is often used as a measurement of co-expressed genes. When the data do not follow multivariate normal distribution, more robust methods, such as the Spearman correlation or the bi-weight mid-correlation, are recommended (Zhang and Horvath, 2005). These methods, however, may not be optimal for “real world” data, which are often influenced by confounding variables, such as the sample processing by separate laboratories in our first experimental sets, a factor known to strongly influence expression measurements. Thus, two genes may have significant correlation not because of shared biological pathways, but simply because the expression measurements were performed by a certain laboratory or technology. Using the model-based method proposed in this paper, we can eliminate the “spurious” co-expression caused by known confounding factors. This is critical when data from multiple experiments are combined in a meta co-expression analysis. For example, consider a confounding factor Z with N(0,1) distribution, and two genes X, Y that are conditionally independent, given Z = z, with distribution N(z,1). It then follows that the Pearson correlation between X and Y is 0.5. As mentioned before, under this scenario, the model-based co-expression test is equivalent to testing whether X and Y have a zero partial correlation, given Z. It is easy to see that here, indeed, the partial correlation between X and Y, given Z, is 0. Thus, we correctly capture the independent relation between X and Y given the confounder Z, and eliminate the spurious correlation.

We recognize that this paper has several limitations. First, the data were collected over a period of 4 years, and two laboratories were used to perform RNAseq in the first experimental set. Although we have developed statistical models to address this shortcoming, it might have negatively affected the power of this study. Second, our results were derived from an informatic analysis of the gene expression data on the basis of RNA sequencing libraries. It will therefore require future experimental validation. This is particularly important as the regulation of mRNA by lncRNA and miRNA is a complex process, involving direct and indirect regulation and chromatin remodeling. For the same reason we did not consider the presence of or absence of miRNA-response elements in gene ontology analysis. Finally, our work is limited to *in vitro* approaches, which may not be fully consistent with changes that occur *in vivo*. In the future, we plan to collect additional data from our experiments and from public databases to strengthen and validate our results.

## Data Availability Statement

The datasets presented in this study can be found in online repositories. The names of the repository/repositories and accession number(s) can be found below: https://www.ncbi.nlm.nih.gov/, PRJNA674312; https://www.ncbi.nlm.nih.gov/, PRJNA674329; https://www.ncbi.nlm.nih.gov/, PRJNA674366; https://www.ncbi.nlm.nih.gov/, PRJNA704383; https://www.ncbi.nlm.nih.gov/, PRJNA704399; https://www.ncbi.nlm.nih.gov/, PRJNA704393.

## Ethics Statement

The studies involving human participants were reviewed and approved by the Institutional Review Board at the University of Pittsburgh. Written informed consent for participation was not required for this study in accordance with the national legislation and the institutional requirements.

## Author Contributions

TC, JFM, and YS: study design, manuscript writing. TC and YS: provided study materials. TC and ZC: data generation and analysis. TC, JFM, YO, OB, and YS: data interpretation. All authors contributed to the article and approved the submitted version.

## Conflict of Interest

YS was a consultant at Illumina, Inc. The remaining authors declare that the research was conducted in the absence of any commercial or financial relationships that could be construed as a potential conflict of interest.

## Footnotes

1 www.qiagen.com/
