# Supplementary material for: RNA Network Interactions During Differentiation of Human Trophoblasts
Source: Front Cell Dev Biol. 2021 Jun 3;9:677981. doi: 10.3389/fcell.2021.677981 (PMC8209545; doi:10.3389/fcell.2021.677981)
Supplement: Supplementary file 1 [file Table_1.PDF]

Supplementary Table 1. Expression changes for the top concordant mRNAs*, ranked by expression change in the two experimental conditions**.

Gene Name	Our data		Vento Tormo Expression values***			Suryawanshi Expression values***		
	Std vs Hpx	Std vs DMSO	Syn	Cyt	Syn/Cyt ratio	Syn	Cyt	Syn/Cyt ratio
Upregulated mRNAs								
LGALS14	6.32	7.34	0.5557	0.0301	18.46	0.4889	0.0067	72.97
DEPDC1B	5.65	5.28	0.2534	0.0952	2.66	0.6247	0.2229	2.80
SLC6A2	4.86	5.21	0.0325	0.0017	19.12	0.0815	0.0173	4.71
CGB3	7.85	8.30	5.6001	0.0342	163.75	NA	NA	NA
LGALS13	6.03	8.22	1.8190	0.0428	42.50	2.7254	0.0116	234.95
HLF	6.43	8.00	0.0061	0.0016	3.81	0.0000	0.0039	0.00
ENDOU	4.51	6.25	0.1811	0.0067	27.03	0.4889	0.0125	39.11
TREML2	6.60	3.59	0.0499	0.0004	124.75	0.0091	0.0000	
INSL4	6.18	6.08	18.6771	2.5957	7.20	37.1517	2.4937	14.90
CGA	7.03	6.35	447.8674	12.8267	34.92	682.8191	18.4303	37.05
CRH	6.35	6.70	0.8838	0.0029	304.76	NA	NA	NA
MFSD2A	4.48	5.26	0.2837	0.0031	91.52	0.0543	0.0193	2.81
HOPX	7.19	7.39	1.7204	0.0164	104.90	2.9156	0.0357	81.67
XKRX	5.95	5.13	0.0129	0.0061	2.11	0.0091	0.0019	4.79
CGB5	8.13	8.04	0.1161	0.0021	55.29	0.1901	0.0087	21.85
CGB8	8.13	7.89	0.2523	0.0054	46.72	0.2897	0.0174	16.65
TCL1B	6.10	6.28	0.3875	0.0139	27.88	0.2535	0.0386	6.57
PSG11	3.88	6.05	0.9611	0.0034	282.68	0.0027	0.0019	1.42
LGALS16	5.24	4.61	5.4549	0.0828	65.88	7.9320	0.0038	2087.37
Downregulated mRNAs								
MAP2	-3.29	-3.59	0.0022	0.0046	0.48	0.0181	0.0096	1.89
COL12A1	-3.27	-3.74	0.0089	0.0058	1.53	0.0000	0.0068	
PPL	-3.13	-4.08	0.0275	0.0156	1.76	0.0000	0.0039	
NPPB	-5.04	-2.55	0.5321	0.0247	21.54	0.0000	0.0395	
C1orf116	-2.66	-4.75	0.0022	0.0003	7.33	NA	NA	NA
SV2B	-4.83	-2.65	0.0000	0.0001		0.0000	0.0000	

* mRNA for comparison were selected as described in the Results section.

** Light orange shading represents mRNAs that were not fully consistent with our data.

*** Analyses was performed using the PlacentaCellEnrich tool (Jain and Tuteja, *Placenta*, 2021;103:164-171, doi:10.1016/j.placenta.2020.10.029) which is based on scRNAseq changes published by Vento-Tormo R., *et al*, (*Nature* 2018;563:347-353, doi: 10.1038/s41586-018-0698-6), and Suryawanshi H. *et al*, (*Sci Adv* 2018;4(10):eaau4788, doi: 10.1126/sciadv.aau4788).
