# Supplementary material for: RNA Network Interactions During Differentiation of Human Trophoblasts
Source: Front Cell Dev Biol. 2021 Jun 3;9:677981. doi: 10.3389/fcell.2021.677981 (PMC8209545; doi:10.3389/fcell.2021.677981)
Supplement: Supplementary file 2 [file Table_2.PDF]

Supplementary Table 2. Species of mRNA that were associated with concordant lncRNA *and* miRNA expression change in the two experimental conditions.

Gene Name	Ensembl Gene D	Std vs Hpx	Std vs DMSO	Description [Source]
HOPX	ENSG00000171476	7.188558195	7.393653751	HOP homeobox [Source:HGNC Symbol;Acc:HGNC:24961]
LGALS13	ENSG00000105198	6.027818874	8.223957751	Galectin 13 [Source:HGNC Symbol;Acc:HGNC:15449]
CGA	ENSG00000135346	7.029780662	6.345096145	Glycoprotein hormones, alpha polypeptide [Source:HGNC Symbol;Acc:HGNC:1885]
ENDOU	ENSG00000111405	4.512867982	6.248070627	Endonuclease, poly(U) specific [Source:HGNC Symbol;Acc:HGNC:14369]
PSG11	ENSG00000243130	3.879819265	6.049952137	Pregnancy specific beta-1-glycoprotein 11 [Source:HGNC Symbol;Acc:HGNC:9516]
SLC30A2	ENSG00000158014	5.132510362	3.958012094	Solute carrier family 30 member 2 [Source:HGNC Symbol;Acc:HGNC:11013]
ADCY5	ENSG00000173175	3.013513955	5.995336615	Aadenylate cyclase 5 [Source:HGNC Symbol;Acc:HGNC:236]
SPRY4	ENSG00000187678	4.984057038	3.73969159	Sprouty RTK signaling antagonist 4 [Source:HGNC Symbol;Acc:HGNC:15533]
KISS1	ENSG00000170498	3.083971996	5.428009696	KiSS-1 metastasis-suppressor [Source:HGNC Symbol;Acc:HGNC:6341]
IGSF5	ENSG00000183067	3.306345842	4.962161263	Immunoglobulin superfamily member 5 [Source:HGNC Symbol;Acc:HGNC:5952]
NECTIN3	ENSG00000177707	3.864055599	3.995756557	Nectin cell adhesion molecule 3 [Source:HGNC Symbol;Acc:HGNC:17664]
SDC1	ENSG00000115884	4.371898204	3.394625165	Syndecan 1 [Source:HGNC Symbol;Acc:HGNC:10658]
PAPPA2	ENSG00000116183	3.173576354	4.477282911	Pappalysin 2 [Source:HGNC Symbol;Acc:HGNC:14615]
NPPB	ENSG00000120937	-5.039718551	-2.545802766	Natriuretic peptide B [Source:HGNC Symbol;Acc:HGNC:7940]
PSG7	ENSG00000221878	4.178642257	3.349957942	Pregnancy specific beta-1-glycoprotein 7 (gene/pseudogene) [Source:HGNC Symbol;Acc:HGNC:9524]
PSG6	ENSG00000170848	5.202780777	2.267207175	Pregnancy specific beta-1-glycoprotein 6 [Source:HGNC Symbol;Acc:HGNC:9523]
POSTN	ENSG00000133110	3.388533028	4.068226592	Periostin [Source:HGNC Symbol;Acc:HGNC:16953]
HSD11B2	ENSG00000176387	4.432179621	2.918730695	Hydroxysteroid 11-beta dehydrogenase 2 [Source:HGNC Symbol;Acc:HGNC:5209]
ADAM12	ENSG00000148848	1.802796609	5.518101449	ADAM metallopeptidase domain 12 [Source:HGNC Symbol;Acc:HGNC:190]
GDF15	ENSG00000130513	2.548582982	4.622594446	Growth differentiation factor 15 [Source:HGNC Symbol;Acc:HGNC:30142]
MNDA	ENSG00000163563	4.164431741	2.788396364	Myeloid cell nuclear differentiation antigen [Source:HGNC Symbol;Acc:HGNC:7183]
MMP7	ENSG00000137673	1.25770468	5.69220674	Matrix metallopeptidase 7 [Source:HGNC Symbol;Acc:HGNC:7174]

Gene Name	Ensembl Gene D	Std vs Hpx	Std vs DMSO	Description [Source]
HK3	ENSG00000160883	4.402314639	2.52847118	Hexokinase 3 [Source:HGNC Symbol;Acc:HGNC:4925]
ELMO1	ENSG00000155849	3.195804706	3.673909592	Engulfment and cell motility 1 [Source:HGNC Symbol;Acc:HGNC:16286]
ADAMTS6	ENSG00000049192	4.127448215	2.661314675	ADAM metallopeptidase with thrombospondin type 1 motif 6 [Source:HGNC Symbol;Acc:HGNC:222]
ADAM19	ENSG00000135074	3.280180837	3.313243061	ADAM metallopeptidase domain 19 [Source:HGNC Symbol;Acc:HGNC:197]
PSG5	ENSG00000204941	4.89003698	1.692362638	Pregnancy specific beta-1-glycoprotein 5 [Source:HGNC Symbol;Acc:HGNC:9522]
STEAP4	ENSG00000127954	2.951839509	3.450547541	STEAP4 metalloreductase [Source:HGNC Symbol;Acc:HGNC:21923]
FZD10	ENSG00000111432	4.331942857	2.044652888	Frizzled class receptor 10 [Source:HGNC Symbol;Acc:HGNC:4039]
FCGR3A	ENSG00000203747	3.733112621	2.411198258	Fc fragment of IgG receptor IIIa [Source:HGNC Symbol;Acc:HGNC:3619]
DPP10	ENSG00000175497	4.292663701	1.791114164	Dipeptidyl peptidase like 10 [Source:HGNC Symbol;Acc:HGNC:20823]
CDKN1C	ENSG00000129757	4.06897294	2.004321794	Cyclin dependent kinase inhibitor 1C [Source:HGNC Symbol;Acc:HGNC:1786]
PSG2	ENSG00000242221	3.381373154	2.608272772	Pregnancy specific beta-1-glycoprotein 2 [Source:HGNC Symbol;Acc:HGNC:9519]
LEP	ENSG00000174697	2.971849415	3.003667251	Leptin [Source:HGNC Symbol;Acc:HGNC:6553]
SLC25A35	ENSG00000125434	3.657688515	2.18093311	Solute carrier family 25 member 35 [Source:HGNC Symbol;Acc:HGNC:31921]
MSR1	ENSG00000038945	3.192267368	2.479202798	Macrophage scavenger receptor 1 [Source:HGNC Symbol;Acc:HGNC:7376]
MAFF	ENSG00000185022	2.367470914	3.191162609	MAF bZIP transcription factor F [Source:HGNC Symbol;Acc:HGNC:6780]
ESRRG	ENSG00000196482	3.062255954	2.448276012	Estrogen related receptor gamma [Source:HGNC Symbol;Acc:HGNC:3474]
CSF2RB	ENSG00000100368	3.018458635	2.485070981	Colony stimulating factor 2 receptor beta Common subunit [Source:HGNC Symbol;Acc:HGNC:2436]
CLIC3	ENSG00000169583	3.697006378	1.680419927	Chloride intracellular channel 3 [Source:HGNC Symbol;Acc:HGNC:2064]
CXCL5	ENSG00000163735	-2.887233951	-2.465778128	C-X-C motif chemokine ligand 5 [Source:HGNC Symbol;Acc:HGNC:10642]
APOC1	ENSG00000130208	3.167497118	2.178652298	Apolipoprotein C1 [Source:HGNC Symbol;Acc:HGNC:607]
SHCBP1	ENSG00000171241	-2.565730094	-2.760349074	SHC binding and spindle associated 1 [Source:HGNC Symbol;Acc:HGNC:29547]
TPPP3	ENSG00000159713	2.480879621	2.620991457	Tubulin polymerization promoting protein family member 3 [Source:HGNC Symbol;Acc:HGNC:24162]

Gene Name	Ensembl Gene D	Std vs Hpx	Std vs DMSO	Description [Source]
SLCO2B1	ENSG00000137491	4.004050074	1.091558567	Solute carrier organic anion transporter family member 2B1 [Source:HGNC Symbol;Acc:HGNC:10962]
CISH	ENSG00000114737	2.600638429	2.379106362	Cytokine inducible SH2 containing protein [Source:HGNC Symbol;Acc:HGNC:1984]
BDKRB2	ENSG00000168398	3.14511925	1.757148649	Bradykinin receptor B2 [Source:HGNC Symbol;Acc:HGNC:1030]
ERVFRD-1	ENSG00000244476	2.098711169	2.686247692	Endogenous retrovirus group FRD member 1 [Source:HGNC Symbol;Acc:HGNC:33823]
AFF1	ENSG00000172493	2.333262349	2.406485848	AF4/FMR2 family member 1 [Source:HGNC Symbol;Acc:HGNC:7135]
RAB11FIP5	ENSG00000135631	1.848629081	2.886440766	RAB11 family interacting protein 5 [Source:HGNC Symbol;Acc:HGNC:24845]
SYT7	ENSG00000011347	3.402365166	1.248619479	Synaptotagmin 7 [Source:HGNC Symbol;Acc:HGNC:11514]
ANK3	ENSG00000151150	3.08064674	1.545215212	Ankyrin 3 [Source:HGNC Symbol;Acc:HGNC:494]
GREM2	ENSG00000180875	2.192953413	2.421273642	Gremlin 2, DAN family BMP antagonist [Source:HGNC Symbol;Acc:HGNC:17655]
SFN	ENSG00000175793	-2.871617954	-1.703711753	Stratifin [Source:HGNC Symbol;Acc:HGNC:10773]
FGL2	ENSG00000127951	3.021951028	1.531832715	Fibrinogen like 2 [Source:HGNC Symbol;Acc:HGNC:3696]
MRC1	ENSG00000260314	3.243013288	1.290117911	Mannose receptor, C type 1 [Source:HGNC Symbol;Acc:HGNC:7228]
ZFAT	ENSG00000066827	3.153794466	1.373308613	Zinc finger and AT-hook domain containing [Source:HGNC Symbol;Acc:HGNC:19899]
SLC19A3	ENSG00000135917	3.003432357	1.509518316	Solute carrier family 19 member 3 [Source:HGNC Symbol;Acc:HGNC:16266]
SPATA13	ENSG00000182957	1.526920697	2.95971298	Spermatogenesis associated 13 [Source:HGNC Symbol;Acc:HGNC:23222]
HTRA1	ENSG00000166033	1.067830741	3.323536771	HtrA serine peptidase 1 [Source:HGNC Symbol;Acc:HGNC:9476]
NOS3	ENSG00000164867	2.918477913	1.458678582	Nitric oxide synthase 3 [Source:HGNC Symbol;Acc:HGNC:7876]
CSF3R	ENSG00000119535	2.832835605	1.497240119	Colony stimulating factor 3 receptor [Source:HGNC Symbol;Acc:HGNC:2439]
BCL2A1	ENSG00000140379	2.12295872	2.200776223	BCL2 related protein A1 [Source:HGNC Symbol;Acc:HGNC:991]
TREM2	ENSG00000095970	2.565642125	1.72787897	Triggering receptor expressed on myeloid cells 2 [Source:HGNC Symbol;Acc:HGNC:17761]
SLC2A11	ENSG00000133460	2.316228341	1.958150505	Solute carrier family 2 member 11 [Source:HGNC Symbol;Acc:HGNC:14239]
MED12L	ENSG00000144893	2.732309761	1.518700729	Mediator complex subunit 12 like [Source:HGNC Symbol;Acc:HGNC:16050]
CYFIP2	ENSG00000055163	-1.914297074	-2.293973533	Cytoplasmic FMR1 interacting protein 2 [Source:HGNC Symbol;Acc:HGNC:13760]

Gene Name	Ensembl Gene D	Std vs Hpx	Std vs DMSO	Description [Source]
RASAL2	ENSG00000075391	2.231752149	1.934752523	RAS protein activator like 2 [Source:HGNC Symbol;Acc:HGNC:9874]
SIAH1	ENSG00000196470	2.543822019	1.618605272	Siah E3 ubiquitin protein ligase 1 [Source:HGNC Symbol;Acc:HGNC:10857]
SNAI1	ENSG00000124216	2.306861951	1.846627738	Snail family transcriptional repressor 1 [Source:HGNC Symbol;Acc:HGNC:11128]
ITGA2	ENSG00000164171	-2.049703982	-2.061455709	Integrin subunit alpha 2 [Source:HGNC Symbol;Acc:HGNC:6137]
FPR3	ENSG00000187474	3.022123448	1.075630677	Formyl peptide receptor 3 [Source:HGNC Symbol;Acc:HGNC:3828]
SOCS2	ENSG00000120833	2.363490459	1.619505531	Suppressor of cytokine signaling 2 [Source:HGNC Symbol;Acc:HGNC:19382]
AQP9	ENSG00000103569	1.218598933	2.744972783	Aquaporin 9 [Source:HGNC Symbol;Acc:HGNC:643]
PAQR7	ENSG00000182749	2.525627944	1.406156094	Progestin and adipoQ receptor family member 7 [Source:HGNC Symbol;Acc:HGNC:23146]
TET1	ENSG00000138336	1.951197637	1.909601814	Tet methylcytosine dioxygenase 1 [Source:HGNC Symbol;Acc:HGNC:29484]
EFS	ENSG00000100842	2.746152906	1.080689225	Embryonal Fyn-associated substrate [Source:HGNC Symbol;Acc:HGNC:16898]
TGM2	ENSG00000198959	1.492282065	2.333456185	Transglutaminase 2 [Source:HGNC Symbol;Acc:HGNC:11778]
TMC5	ENSG00000103534	2.25921475	1.559820412	Transmembrane channel like 5 [Source:HGNC Symbol;Acc:HGNC:22999]
TTC7B	ENSG00000165914	2.331948223	1.452959639	Tetratricopeptide repeat domain 7B [Source:HGNC Symbol;Acc:HGNC:19858]
LPL	ENSG00000175445	1.148917883	2.605057591	Lipoprotein lipase [Source:HGNC Symbol;Acc:HGNC:6677]
CD36	ENSG00000135218	2.02894203	1.694248016	CD36 molecule [Source:HGNC Symbol;Acc:HGNC:1663]
CDC25A	ENSG00000164045	-1.459317067	-2.250400271	Cell division cycle 25A [Source:HGNC Symbol;Acc:HGNC:1725]
ZNF488	ENSG00000265763	1.636172323	2.027142278	Zinc finger protein 488 [Source:HGNC Symbol;Acc:HGNC:23535]
VSIG4	ENSG00000155659	2.391067324	1.243511554	V-set and immunoglobulin domain containing 4 [Source:HGNC Symbol;Acc:HGNC:17032]
ALDH1A1	ENSG00000165092	1.811258913	1.793796295	Aldehyde dehydrogenase 1 family member A1 [Source:HGNC Symbol;Acc:HGNC:402]
FOSB	ENSG00000125740	1.667380248	1.934300295	FosB proto-oncogene, AP-1 transcription factor subunit [Source:HGNC Symbol;Acc:HGNC:3797]
ST3GAL5	ENSG00000115525	1.575395964	1.998079343	ST3 beta-galactoside alpha-2,3-Sialyltransferase 5 [Source:HGNC Symbol;Acc:HGNC:10872]

Gene Name	Ensembl Gene D	Std vs Hpx	Std vs DMSO	Description [Source]
ISM2	ENSG00000100593	1.424018467	2.131770269	Isthmin 2 [Source:HGNC Symbol;Acc:HGNC:23176]
CHST3	ENSG00000122863	2.273308374	1.273962496	Carbohydrate sulfotransferase 3 [Source:HGNC Symbol;Acc:HGNC:1971]
GBP5	ENSG00000154451	1.731160669	1.806729961	Guanylate binding protein 5 [Source:HGNC Symbol;Acc:HGNC:19895]
C1orf115	ENSG00000162817	2.155233729	1.361471943	Chromosome 1 open reading frame 115 [Source:HGNC Symbol;Acc:HGNC:25873]
ANKRD36	ENSG00000135976	-2.116932117	-1.389024606	Ankyrin repeat domain 36 [Source:HGNC Symbol;Acc:HGNC:24079]
DHCR7	ENSG00000172893	1.524987385	1.969236056	7-dehydrocholesterol reductase [Source:HGNC Symbol;Acc:HGNC:2860]
FAM184A	ENSG00000111879	2.182666605	1.311483144	Family with sequence similarity 184 member A [Source:HGNC Symbol;Acc:HGNC:20991]
BCORL1	ENSG00000085185	1.593814609	1.843054856	BCL6 corepressor-like 1 [Source:HGNC Symbol;Acc:HGNC:25657]
HAVCR2	ENSG00000135077	2.179385763	1.237247238	Hepatitis A virus cellular receptor 2 [Source:HGNC Symbol;Acc:HGNC:18437]
LDLR	ENSG00000130164	1.439047155	1.977000218	Low density lipoprotein receptor [Source:HGNC Symbol;Acc:HGNC:6547]
GLUL	ENSG00000135821	2.328496966	1.08148603	Glutamate-ammonia ligase [Source:HGNC Symbol;Acc:HGNC:4341]
TMTC2	ENSG00000179104	-1.753568598	-1.654197395	Transmembrane and tetratricopeptide repeat containing 2 [Source:HGNC Symbol;Acc:HGNC:25440]
GALK1	ENSG00000108479	-1.870374273	-1.53159789	Galactokinase 1 [Source:HGNC Symbol;Acc:HGNC:4118]
CTSK	ENSG00000143387	1.338190959	2.052592095	Cathepsin K [Source:HGNC Symbol;Acc:HGNC:2536]
CECR2	ENSG00000099954	1.329933976	2.03618	CECR2, histone acetyl-lysine reader [Source:HGNC Symbol;Acc:HGNC:1840]
SVEP1	ENSG00000165124	1.574077491	1.752057689	Sushi, von Willebrand factor type A, EGF and pentraxin domain containing 1 [Source:HGNC Symbol;Acc:HGNC:15985]
HIST1H1C	ENSG00000187837	1.584160964	1.72411835	Histone cluster 1 H1 family member c [Source:HGNC Symbol;Acc:HGNC:4716]
PRNP	ENSG00000171867	-2.02863449	-1.214751622	Prion protein [Source:HGNC Symbol;Acc:HGNC:9449]
LY86	ENSG00000112799	2.210394069	1.010485129	Lymphocyte antigen 86 [Source:HGNC Symbol;Acc:HGNC:16837]
PPARD	ENSG00000112033	2.001259733	1.158182185	Peroxisome proliferator activated receptor delta [Source:HGNC Symbol;Acc:HGNC:9235]
FAM198B	ENSG00000164125	2.017387383	1.123914016	Family with sequence similarity 198 member B [Source:HGNC Symbol;Acc:HGNC:25312]

Gene Name	Ensembl Gene D	Std vs Hpx	Std vs DMSO	Description [Source]
PECAM1	ENSG00000261371	1.212792691	1.890625421	Platelet and endothelial cell adhesion molecule 1 [Source:HGNC Symbol;Acc:HGNC:8823]
NID1	ENSG00000116962	1.912130277	1.176142232	Nidogen 1 [Source:HGNC Symbol;Acc:HGNC:7821]
MMP9	ENSG00000100985	1.196542075	1.889546783	Matrix metallopeptidase 9 [Source:HGNC Symbol;Acc:HGNC:7176]
IQSEC1	ENSG00000144711	1.425960998	1.657118469	IQ motif and Sec7 domain 1 [Source:HGNC Symbol;Acc:HGNC:29112]
ZNF114	ENSG00000178150	1.00334692	2.071342869	Zinc finger protein 114 [Source:HGNC Symbol;Acc:HGNC:12894]
OVCH2	ENSG00000183378	1.42580405	1.634629618	Ovochymase 2 (gene/pseudogene) [Source:HGNC Symbol;Acc:HGNC:29970]
GALM	ENSG00000143891	1.472428615	1.531601008	Galactose mutarotase [Source:HGNC Symbol;Acc:HGNC:24063]
SYNJ1	ENSG00000159082	1.535833991	1.460570147	Synaptojanin 1 [Source:HGNC Symbol;Acc:HGNC:11503]
PLCB2	ENSG00000137841	1.592758048	1.389034594	Phospholipase C beta 2 [Source:HGNC Symbol;Acc:HGNC:9055]
NHSL1	ENSG00000135540	1.33596615	1.57053795	NHS like 1 [Source:HGNC Symbol;Acc:HGNC:21021]
MILR1	ENSG00000271605	1.552372659	1.316186744	Mast cell immunoglobulin like receptor 1 [Source:HGNC Symbol;Acc:HGNC:27570]
PDE4B	ENSG00000184588	1.651100505	1.195874005	Phosphodiesterase 4B [Source:HGNC Symbol;Acc:HGNC:8781]
INSIG1	ENSG00000186480	1.64435234	1.181002614	Insulin induced gene 1 [Source:HGNC Symbol;Acc:HGNC:6083]
DIP2B	ENSG00000066084	1.647884434	1.124293188	Disco interacting protein 2 homolog B [Source:HGNC Symbol;Acc:HGNC:29284]
RBBP6	ENSG00000122257	1.389813203	1.376980259	RB binding protein 6, ubiquitin ligase [Source:HGNC Symbol;Acc:HGNC:9889]
CTSS	ENSG00000163131	1.368737702	1.366272192	Cathepsin S [Source:HGNC Symbol;Acc:HGNC:2545]
MORC4	ENSG00000133131	1.41519042	1.277971272	MORC family CW-type zinc finger 4 [Source:HGNC Symbol;Acc:HGNC:23485]
FARP1	ENSG00000152767	-1.400986206	-1.282039774	FERM, ARH/RhoGEF and pleckstrin domain protein 1 [Source:HGNC Symbol;Acc:HGNC:3591]
TYROBP	ENSG00000011600	1.633687594	1.042003375	TYRO protein tyrosine kinase binding protein [Source:HGNC Symbol;Acc:HGNC:12449]
TUBB6	ENSG00000176014	-1.31197912	-1.33388855	Tubulin beta 6 class V [Source:HGNC Symbol;Acc:HGNC:20776]
FNIP2	ENSG00000052795	1.288250367	1.339973501	Folliculin interacting protein 2 [Source:HGNC Symbol;Acc:HGNC:29280]
ARHGAP4	ENSG00000089820	1.154366577	1.403827588	Rho GTPase activating protein 4 [Source:HGNC Symbol;Acc:HGNC:674]
TGFBI	ENSG00000120708	1.528382997	1.022338956	Transforming growth factor beta induced [Source:HGNC Symbol;Acc:HGNC:11771]

Gene Name	Ensembl Gene D	Std vs Hpx	Std vs DMSO	Description [Source]
TMEM94	ENSG00000177728	1.215162412	1.327272785	Transmembrane protein 94 [Source:HGNC Symbol;Acc:HGNC:28983]
NR6A1	ENSG00000148200	1.378936789	1.130504333	Nuclear receptor subfamily 6 group A member 1 [Source:HGNC Symbol;Acc:HGNC:7985]
DOCK2	ENSG00000134516	1.440377752	1.065429684	Dedicator of cytokinesis 2 [Source:HGNC Symbol;Acc:HGNC:2988]
TTBK2	ENSG00000128881	1.019215611	1.461005932	Tau tubulin kinase 2 [Source:HGNC Symbol;Acc:HGNC:19141]
C3AR1	ENSG00000171860	1.168838236	1.266222256	Complement C3a receptor 1 [Source:HGNC Symbol;Acc:HGNC:1319]
THEMIS2	ENSG00000130775	1.22562319	1.186933879	Thymocyte selection associated family member 2 [Source:HGNC Symbol;Acc:HGNC:16839]
SERPINA1	ENSG00000197249	1.007373531	1.31788943	Serpin family A member 1 [Source:HGNC Symbol;Acc:HGNC:8941]
IRF8	ENSG00000140968	1.148245744	1.167246152	Interferon regulatory factor 8 [Source:HGNC Symbol;Acc:HGNC:5358]
HMGCS1	ENSG00000112972	1.037268334	1.205459802	3-hydroxy-3-methylglutaryl-CoA synthase 1 [Source:HGNC Symbol;Acc:HGNC:5007]
PTPRC	ENSG00000081237	1.1399758	1.064216766	Protein tyrosine phosphatase, receptor type C [Source:HGNC Symbol;Acc:HGNC:9666]
TSPO	ENSG00000100300	1.080766063	1.101788101	Translocator protein [Source:HGNC Symbol;Acc:HGNC:1158]
TMSB4X	ENSG00000205542	1.07918125	1.032654767	Thymosin beta 4, X-linked [Source:HGNC Symbol;Acc:HGNC:11881]
APOE	ENSG00000130203	1.015960041	1.087985816	Apolipoprotein E [Source:HGNC Symbol;Acc:HGNC:613]
