# Supplementary material for: RNA Network Interactions During Differentiation of Human Trophoblasts
Source: Front Cell Dev Biol. 2021 Jun 3;9:677981. doi: 10.3389/fcell.2021.677981 (PMC8209545; doi:10.3389/fcell.2021.677981)
Supplement: Supplementary file 3 [file Image_1.PDF]

A

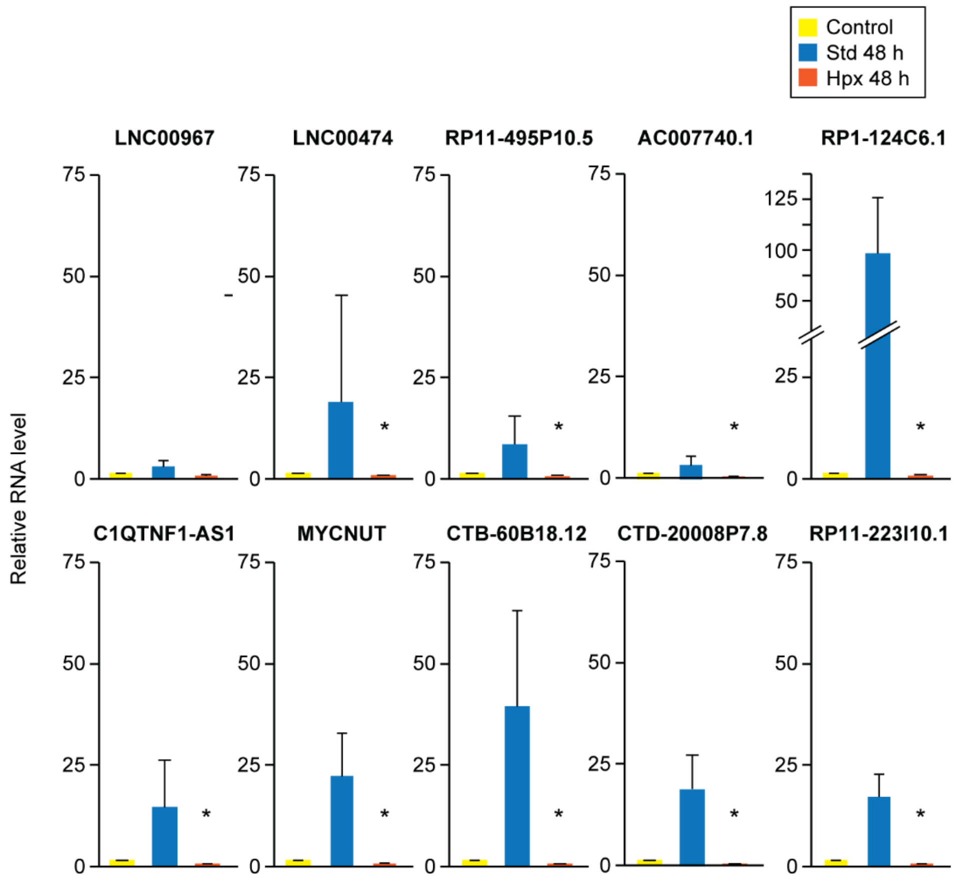

B

List of primers used in the experiments.

lncRNA	Forward primer	Reverse primer
LINC00967	TGCACTGTCAGCTTCCCTAC	TCACAAGGTGAAGTCCCACA
LINC00474	ACTGGGGCTCTTCCTTCAGT	CATAGGTCACATCCCTGCAA
RP11-495P10.5	CGACCTAACCTCGTCTGGAG	GCCATTGTCGTAGGCTTTGT
AC007740.1	AAGAACCACCACCTGCAAAC	CGCAATTCTGTAGGCGAAGT
RP1-124C6.1	GCTGTCCTGATGCTCTTCCT	GCGGTGTCCTTTTGAGAATC
C1QTNF1-AS1	TCTAAAGTGGGCAGGGTAGC	CTCCGAAGCTCTGTGGAGAC
MYCNUT	GAGCACCAAAGCACACACAG	TCCACTCTTCATGATCCATCC
CTB-60B18.12	TGAACCCCATCTCCCAGATA	GGGTGGAGGAGGAGAGAAAC
CTD-2008P7.8	CATCTACCCAGCCGTTTAGC	TAGGAGGTGGAGTGGTCAGC
RP11-223I10.1	CCTTCCAATTGCCCAAAATA	ATTGGTCTGGGTGGACTGAG

Supplementary Figure 1. The impact of hypoxia on the expression lncRNA in PHT cells. These data were used to validate the expression changes in lncRNAs, detected by RNAseq. **A.** RT-q PCR of 10 selected lncRNAs in PHT cells. The cells were harvested either 4 h after plating (control) or after 48 h of culture in standard (Std) conditions or after 48 h of culture in hypoxia (Hpx), which hinders differentiation. The experiments include cells from three different placentas, each assayed separately. * denotes p<0.05. **B.** A list of the primers used in the experiments.
