# Supplementary material for: RNA Network Interactions During Differentiation of Human Trophoblasts
Source: Front Cell Dev Biol. 2021 Jun 3;9:677981. doi: 10.3389/fcell.2021.677981 (PMC8209545; doi:10.3389/fcell.2021.677981)
Supplement: Supplementary file 4 [file Image_2.PDF]

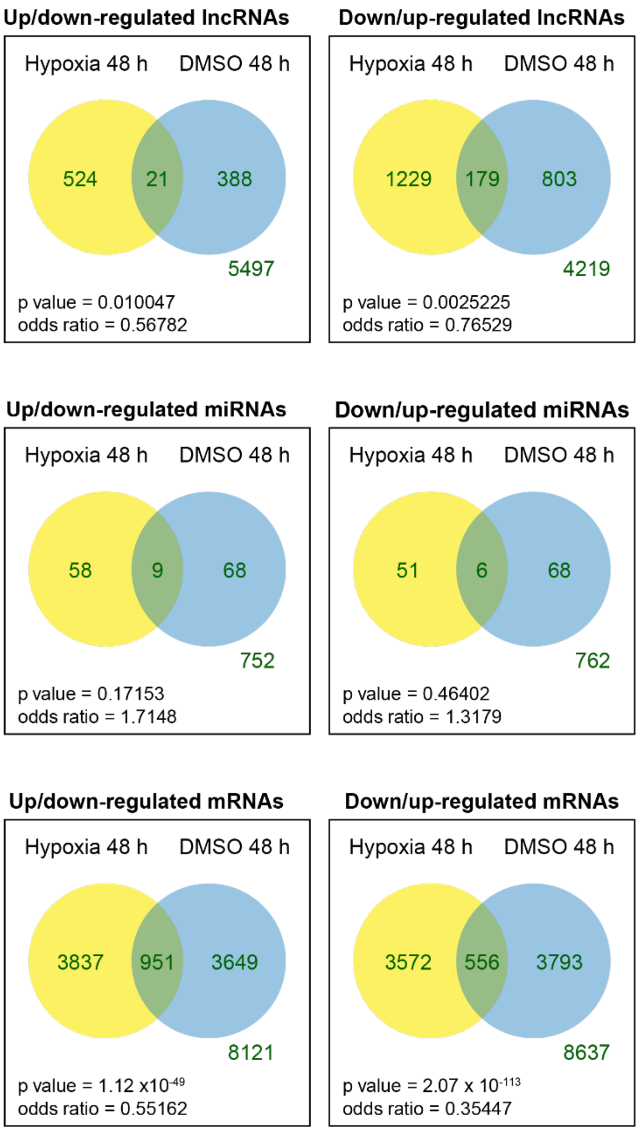


**Supplementary Figure 2. Venn Diagram: mRNAs, lncRNAs, miRNAs with inconsistent pattern**. Shown are differences between the 2 main experimental paradigms. Fisher exact tests were used to determine the differences in RNA expression changes (up- or downregulation) between the two experimental sets. The odds ratio and p-values for differences are depicted within each frame.
