# Supplementary material for: RNA Network Interactions During Differentiation of Human Trophoblasts
Source: Front Cell Dev Biol. 2021 Jun 3;9:677981. doi: 10.3389/fcell.2021.677981 (PMC8209545; doi:10.3389/fcell.2021.677981)
Supplement: Supplementary file 5 [file Image_3.PDF]

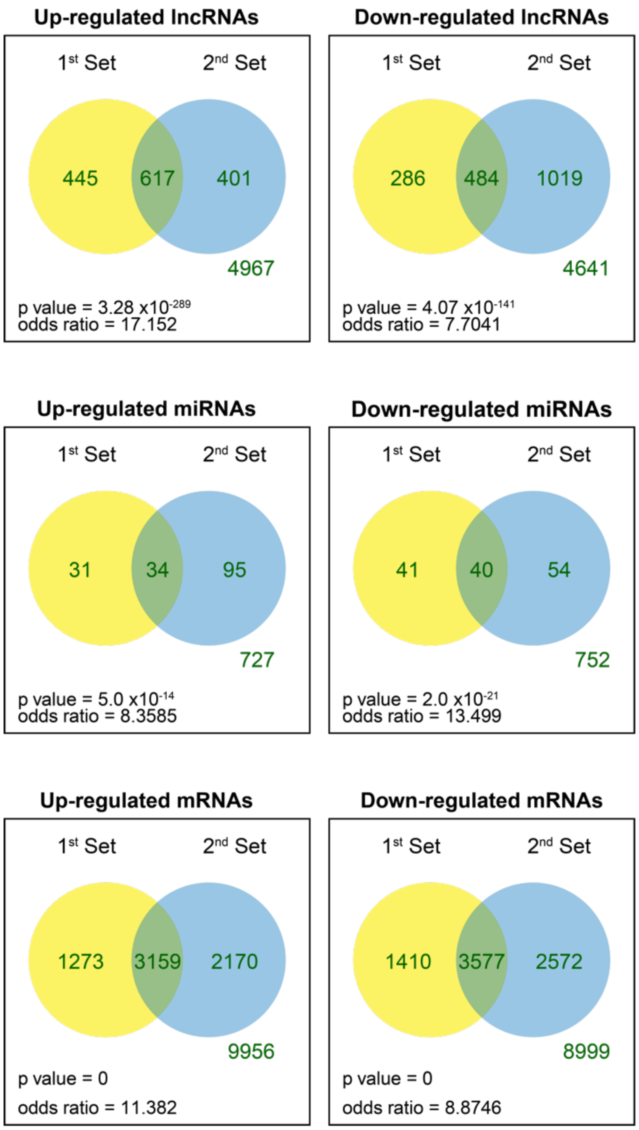

Supplementary Figure 3. A Venn diagram of expressed RNA expression changes between time 0 h and 48 h in standard conditions across the two experimental sets. Fisher exact tests were used to determine the similarity in RNA expression changes (up- or downregulation) between the two experimental sets. The odds ratio and p-values for data similarity are depicted within each frame.
