# Supplementary material for: RNA Network Interactions During Differentiation of Human Trophoblasts
Source: Front Cell Dev Biol. 2021 Jun 3;9:677981. doi: 10.3389/fcell.2021.677981 (PMC8209545; doi:10.3389/fcell.2021.677981)
Supplement: Supplementary file 6 [file Image_4.PDF]

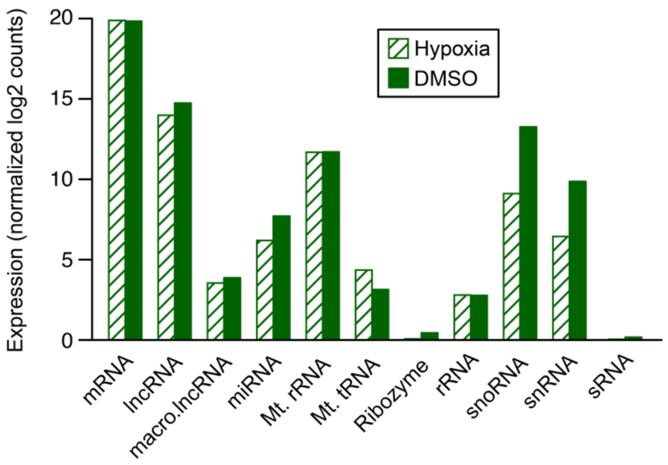

Supplementary Figure 4: **The expression of "other RNAs" (Figure 2) in the two experimental sets.** The X-axis represents selected RNAs species, and the Y-axis represents RNA levels, expressed as normalized log2 RNA counts, for the two experimental conditions. Mt. -mitochondrial.
